# Integrating health equity in artificial intelligence for public health in Canada: a rapid narrative review

**DOI:** 10.3389/fpubh.2025.1524616

**Published:** 2025-03-18

**Authors:** Samantha Ghanem, Marielle Moraleja, Danielle Gravesande, Jennifer Rooney

**Affiliations:** Public Health Agency of Canada, Ottawa, ON, Canada

**Keywords:** artificial intelligence, health equity, public health, AI biases, AI ethics

## Abstract

**Introduction:**

The application of artificial intelligence (AI) in public health is rapidly evolving, offering promising advancements in various public health settings across Canada. AI has the potential to enhance the effectiveness, precision, decision-making, and scalability of public health initiatives. However, to leverage AI in public health without exacerbating inequities, health equity considerations must be addressed. This rapid narrative review aims to synthesize health equity considerations related to AI application in public health.

**Methods:**

A rapid narrative review methodology was used to identify and synthesize literature on health equity considerations for AI application in public health. After conducting title/abstract and full-text screening of articles, and consensus decision on study inclusion, the data extraction process proceeded using an extraction template. Data synthesis included the identification of challenges and opportunities for strengthening health equity in AI application for public health.

**Results:**

The review included 54 peer-review articles and grey literature sources. Several health equity considerations for applying AI in public health were identified, including gaps in AI epistemology, algorithmic bias, accessibility of AI technologies, ethical and privacy concerns, unrepresentative training datasets, lack of transparency and interpretability of AI models, and challenges in scaling technical skills.

**Conclusion:**

While AI has the potential to advance public health in Canada, addressing equity is critical to preventing inequities. Opportunities to strengthen health equity in AI include implementing diverse AI frameworks, ensuring human oversight, using advanced modeling techniques to mitigate biases, fostering intersectoral collaboration for equitable AI development, and standardizing ethical and privacy guidelines to enhance AI governance.

## 1 Introduction

The use of artificial intelligence (AI) in public health is rapidly evolving, offering promising advancements in surveillance, research, policy, and programming that enhance effectiveness, precision, decision-making, and scalability (1). AI refers to technologies that enable computers and machines to simulate human intelligence and problem-solving capabilities, employing methods such as computer vision, natural language processing, and machine learning (ML) (2–5). ML, a field of AI, develops models for prediction and clustering by using a learning dataset, which includes data split into training, validation, and test sets, to train and evaluate algorithms (6). The training data is a specific subset of the learning dataset used directly to teach the model, enabling it to recognize patterns and relationships within the data (4).

Health equity aims to create equal opportunities for all by eliminating unfair, avoidable differences in health status and life expectancy between socially advantaged and disadvantaged groups, involving efforts both within and beyond the health sector (7). As AI application in public health increases, it is crucial to address health equity considerations to ensure its effective use, without exacerbating existing inequities among priority populations (8, 9). Priority populations are communities who are placed at greater risk of adverse health outcomes due to overlapping and intersecting systems of oppression, including persons with disabilities, 2SLGBTQI+ communities, racialized people, and First Nations, Inuit, and Métis, among others (10).

The use of AI in public health raises several equity concerns, including algorithmic bias, transparency and interpretability of models, and accessibility of AI technologies, bringing into question significant ethical, regulatory, and privacy concerns (11, 12). Addressing these issues is crucial to ensuring that AI contributes to improving public health outcomes for all, rather than reinforcing existing societal inequities and vulnerabilities. Therefore, this review aims to synthesize health equity considerations in the application of AI in public health, offering timely insights as AI adoption accelerates.

## 2 Methods

### 2.1 Design

A rapid narrative review was conducted to synthesize the evidence on health equity considerations related to AI in public health. A rapid review is a form of knowledge synthesis that accelerates the process of conducting a traditional review to produce evidence in a resource-efficient manner (13). The rapid narrative review protocol was informed by Arksey and O’Malley’s (14) methodological framework and Peters et al.’s (15) updated guidelines and consists of the following steps: scoping, searching, screening, and data extraction and analysis.

### 2.2 Scoping

A preliminary search using the terms AI, public health, and health equity was conducted in the Government of Canada’s Health Library. Eligibility criteria was established using the populations, issue, context, outcomes (PICO) framework, with the goal of identifying studies that met two main criteria: (1) the use of AI in a public health setting and (2) the explicit or implicit integration of health equity considerations (e.g., biases, privacy, transparency, discrimination). English articles published in Canada between 2014 and 2024 specific to AI application in public health settings were included, while studies published in the same time frame conducted in healthcare, telemedicine, or biomedical settings were excluded for lacking sufficient evidence on health equity related to AI in public health.

### 2.3 Search strategy

A librarian from the Government of Canada’s Health Library developed and executed the search strategy. The search was conducted in Medline and Embase for studies published between January 1, 2014 and June 20, 2024, using search strings related to AI, public health, and health equity (Appendix A). Results were imported into Zotero for management. A grey literature search using search strings related to AI, public health, and health equity was also conducted between May 21, 2024 to July 24, 2024, through targeted website searches, browser keyword searches (including the first 10 pages of Google Scholar), and by identifying literature from cited results.

### 2.4 Screening

Screening was conducted in two phases. In level one, titles and abstracts were screened in Covidence (16), with eligible studies moving to full-text review in level two. To ensure inter-rater reliability, MM and SG screened the first 20% of titles and abstracts, resolving discrepancies through discussion. MM then screened the remaining titles and abstracts, discussing ambiguous articles with SG. For level two, MM and DG conducted full-text reviews of the first 10 studies in duplicate, resolving any discrepancies through discussion. MM screened the remaining full-text articles, and any ambiguities were resolved through discussion with SG and DG.

### 2.5 Data extraction and analysis

**Figure 1:**
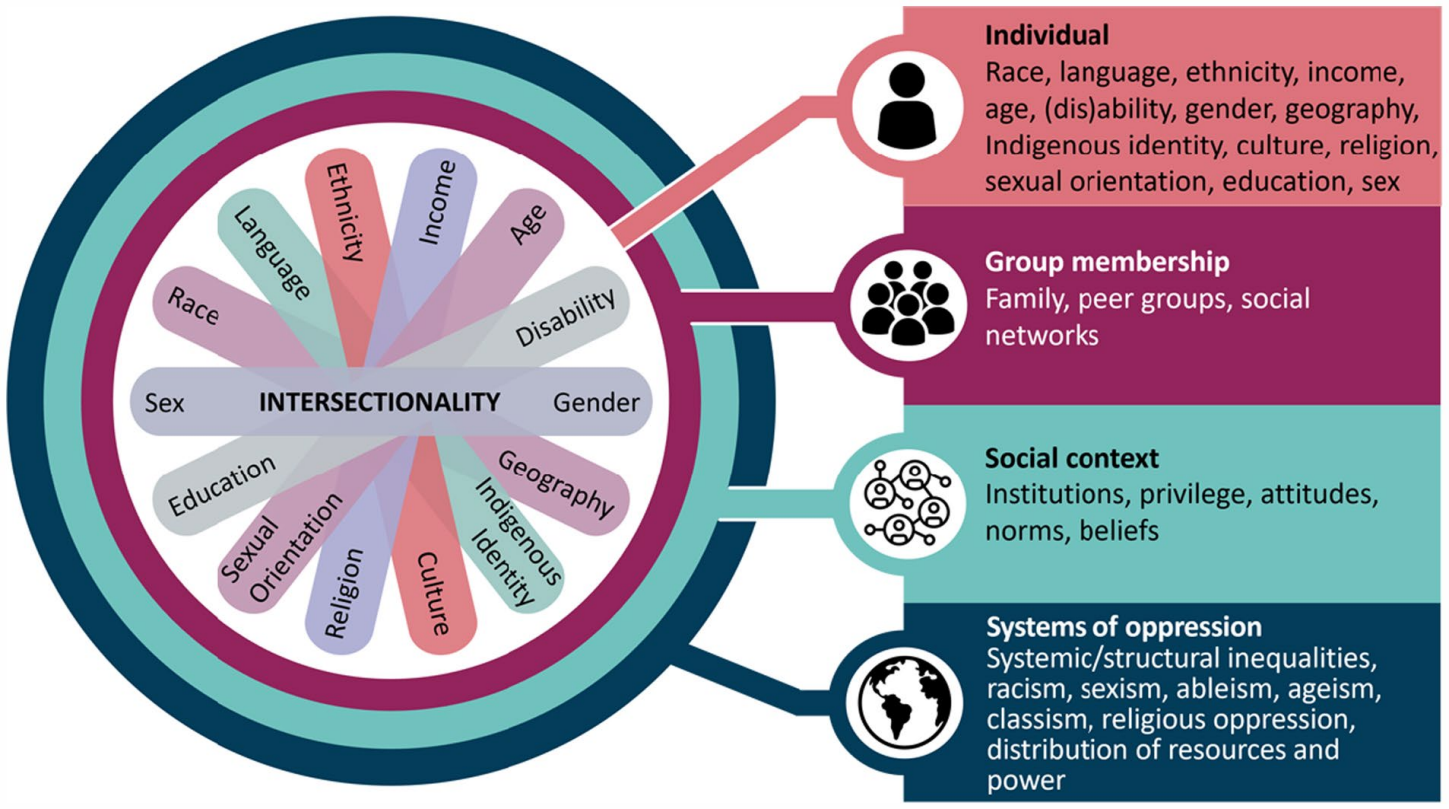
SGBA Plus intersectionality wheel and flower. This figure illustrates some of the determinants of health that intersect to shape our experiences and realities. A figure depicting social identities is centered within a concentric circle of four layers. From the center of the circle, and moving outwards, the figure describes intersectionality considerations related to individual-level factors, group membership, social context, and systems of oppressions, that is, from individual identities to increasingly broad levels of influence. At the center, seven oblong shapes of differing colors overlap and fan out. At the end of each oblong an individual social identity is named. The individual social identities named on the figure are sex, race, language, ethnicity, income, age, (dis) ability, gender, geography, Indigenous identity, culture, religion, sexual orientation, and education. The second layer of the circle directly surrounding the individual social identities is “Group membership” with the following examples listed: family, peer groups, and social networks. The third layer of the circle surrounding group membership is “Social context” with the following examples listed: institutions, privilege, attitudes, norms, and beliefs. The outermost level, which surrounds social context is “systems of oppression,” with the following examples listed: systemic/structural inequalities, racism, sexism, ableism, ageism, classism, religious oppression, and distribution of resources and power. Reprinted with permission from “Integrating Health Equity into Funding Proposals: A Guide for Applicants”, Canada. ca, 2024.

Data was extracted on study characteristics (first author, publication year, study objective, design, and location), public health setting, and health equity considerations. Health equity considerations were based on the Government of Canada’s Health Portfolio Sex- and Gender-Based Analysis Plus (SGBA Plus), an intersectional, analytical framework, which outlines health equity considerations at various levels, including individual social identities, group membership, social context, and systems of oppression (Figure 1) (17). Originating from Black feminist studies, intersectionality theory acknowledges that systems and structures of power, such as racism, sexism, classism, and ableism, do not operate independently (18). Instead, they intersect and overlap, to create distinct experiences of privilege and discrimination based on one’s multi-dimensional identities and social positionality, such as race, ethnicity, gender, socio-economic status, and disability (18). MM extracted data from all studies with guidance from SG. A descriptive analysis was conducted to synthesize key health equity considerations for AI in public health.

## 3 Results

### 3.1 Search results

A total of 263 articles were identified in a Canadian-based search on equity considerations in the use of AI within public health policy, programs, research, surveillance, and initiatives. This total includes 56 references from other sources, including grey literature and citation searching, as well as seven additional relevant articles identified on July 12, 2024, when SG and MM refined the search strategy with the librarian.

After removing 43 duplicates and 1 redacted article, 233 articles were excluded during level one screening for not meeting eligibility, with common reasons being their focus on healthcare settings, advanced statistical methods without AI, or different use of the acronym “AI” (e.g., Alaskan Indian or American Indian). In level two screening, 32 more articles were excluded for not addressing health equity considerations related to AI use in public health. In total, 54 peer-review articles and grey literature articles were included for analysis.

**Figure 2:**
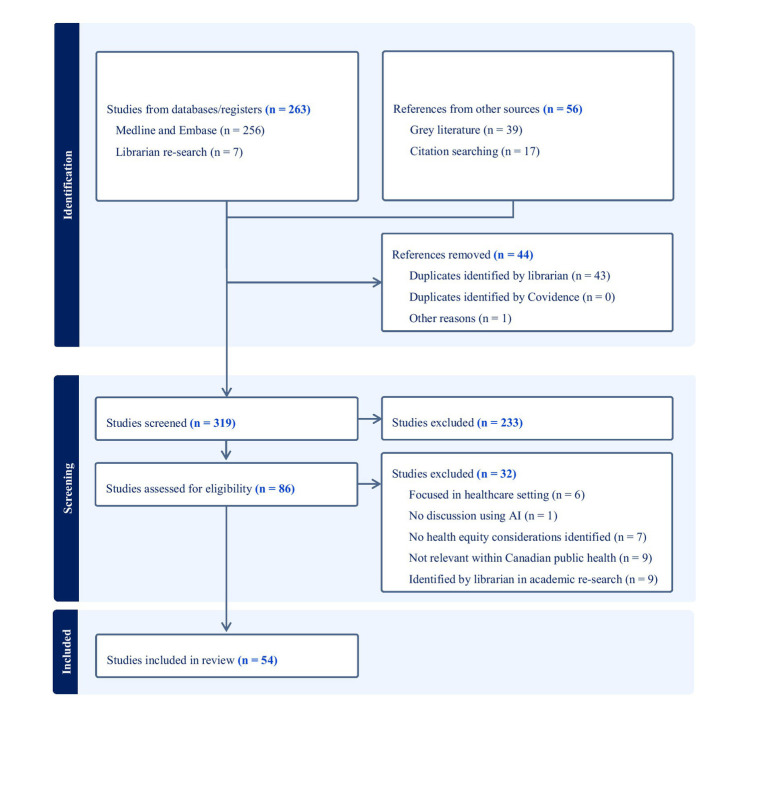
PRISMA flowchart that displays the process of search and selection of studies.

The review was conducted according to the PRISMA Flow Diagram derived from Covidence (Figure 2) (19). Most studies highlighted health equity considerations related to social context and systems of oppression when using AI in public health (Appendix B).

### 3.2 Challenges in integrating health equity in AI use for public health

#### 3.2.1 Systems of oppression

##### 3.2.1.1 Epistemology within AI

Mainstream AI is often grounded in Western epistemology, limiting its scope to specific frameworks and datasets (20, 21). For example, AI systems are typically better at processing English data compared to other languages, which restricts the diversity of knowledge and perspectives interpreted (22, 23). This individualistic approach, rooted in Western epistemology, also fails to consider community-based outcomes or the human-AI relationship (20, 21, 24, 25). Bavli and Galea (26) noted that generative AI, like ChatGPT tends to adopt a pro-environmental, left-libertarian ideology, which can lead to biased outputs. Moreover, Indigenous communities and research organizations have found that AI systems neglect Indigenous knowledge systems, such as Two-Eyed Seeing or the Seventh Generation Principle (20, 27). This risks perpetuating biases that can further marginalize priority populations, such as Indigenous Peoples, racialized communities, 2SLGBTQI+ communities, and women (8, 22, 28–30).

##### 3.2.1.2 Biases throughout the AI lifecycle

Due to gaps in AI epistemology, biases exist throughout the AI lifecycle. Studies highlight various biases in AI, including omitted variable bias, sampling bias, ascertainment bias, selection bias, and measurement errors (9, 31–33). These biases can emerge during the development of AI and its algorithms, often resulting from limited datasets, implicit developer bias, or intentional programming (6, 9, 12, 28, 29, 32, 34–40). Since bias can manifest at any stage of the lifecycle, it complicates the identification of biased outputs and the methods by which biases were introduced into the system (37, 41).

Biases in AI often reflect systems of oppression, such as racism, sexism, and socioeconomic discrimination, exacerbating gender and racial inequalities (6, 8, 12, 39, 41). For instance, Luccioni and Bengio (8) noted a 34.4% error rate in facial recognition technologies for darker-skinned females compared to lighter-skinned males. Text embedding in AI has also been reported to perpetuate gender stereotypes by associating certain statements or terms to a gender (8, 22, 41–43). Unaddressed biases in AI can further exacerbate health inequities in public health decision-making, research, surveillance, resource allocation, and contact tracing (22, 28, 34).

##### 3.2.1.3 Digital divide

Inequitable access to AI technology and its impacts, known as the “digital divide,” deepens systemic inequities (44, 45). Groups commonly lacking access to AI technologies include those in northern, rural, and remote regions, those with lower socioeconomic status, and older adults (6, 33, 45, 46). For example, public health systems in remote areas, such as Yukon, face limited AI resources and workforce capacity, resulting in outdated technologies that delay public health decision-making, precise surveillance, and early interventions (33, 39, 45, 46).

The data used to train AI models can also reflect these disparities, as available data often excludes priority populations, such as Indigenous Peoples, racialized communities, older adults, and those living on low incomes with limited access to technology (12, 23, 47–49). This exclusion limits representation and access to AI knowledge and skills for priority populations (39, 50).

Additionally, AI technologies contribute to ecological burden disproportionately impacting priority populations who are environmentally vulnerable to climate-related events (22, 41, 44, 51). Furthermore, companies often engaging in inequitable labor practices to rapidly develop AI technologies, exacerbate and perpetuate socio-economic inequalities in the Global South and perpetuate colonial practices through resource exploitation and control (22, 39, 41). This deepens the “digital divide” and worsens health inequities among priority populations.

#### 3.2.2 Social context

##### 3.2.2.1 Institutional lag

Institutional lag has occurred as AI implementation in public health has outpaced the development of ethical and privacy guidelines (8, 12, 35, 52). Inconsistencies and non-standardized guidelines across institutions are driven by differing cultural and societal values (8, 9, 31, 53, 54). This has resulted in the use of competitive AI technologies that raise ethical and privacy concerns, such as unregulated data mining, copyright issues, and security breaches, which can perpetuate health inequities (22, 27, 30, 34, 46, 55–57). For instance, Gómez-Ramírez et al. (46) demonstrated that facial recognition technology and “immunity passports” during COVID-19 compromised privacy, equity, and human rights, restricted movement and access to services, and eroded public trust, ultimately undermining other effective public health interventions (29). The lack of accountability mechanisms worsens this mistrust, as there are no enforceable regulations to hold institutions liable for AI-related harms (26, 29, 46, 53, 58).

##### 3.2.2.2 Limited data sets

A key challenge in advancing AI in public health is the limited availability of representative data sets for training and use (6, 22, 58, 59). Available data sets often exclude priority populations, including Indigenous Peoples, racialized communities, 2SLGBTQI+ communities, women, and varying age groups (30, 32, 37). Institutional gatekeeping, high costs, and lack of anonymization further restrict access to robust disaggregated data (25, 26, 32, 38, 42, 58–61). Consequently, organizations often rely on lower quality data that fail to capture socio-demographic factors, leading to inaccurate AI outcomes and the potential for bias (22, 42, 52). For example, Gurevich et al. (6) found that training data often reflects predominately White populations, excluding race-based data. Additionally, studies using natural language processing (NLP) often overlooked older adults, youth, and non-English speakers, limiting the use of disaggregated data (23, 25, 31, 47–49). The omission of social determinants of health from training data, such as socioeconomic status, disability, gender, further limits equitable AI (6, 34). Ethical and privacy concerns, regarding the misuse of disaggregated data, contribute to public mistrust, deterring priority populations from participating in data collection, which perpetuates health inequities (46).

##### 3.2.2.3 AI outputs

It is important to consider the outputs of AI in public health settings, as its lack of interpretability, often described as a “black box,” obscures the reasoning behind results (9, 32, 34, 41, 42, 58, 59). This lack of transparency can perpetuate biases, stereotypes, and discrimination, hindering equitable public health decision-making and implementation. Furthermore, inadequate training data and algorithms can lead to “hallucinations,” which are fabricated data that contribute to misinformation and public mistrust (22, 26, 41). For example, generative AI, including Chat-GPT, has been shown to provide inaccurate information about the COVID-19 pandemic, vaccines, and other public health-related topics (26, 41, 43, 62–64).

#### 3.2.3 Group membership

##### 3.2.3.1 Technical skills

Digital literacy and technical skills, combined with public health expertise, are essential for understanding AI systems and preventing adverse outcomes among priority populations. Barriers such as inadequate training, limited funding, lack of infrastructure, and insufficient resources hinder public health professionals from enhancing their technical skills, potentially leading to health equity gaps (22, 32, 33, 38, 46). This leads practitioners to rely on AI developers who may lack public health expertise and hold implicit biases (12, 32, 35, 58). A lack of technical skills, coupled with a non-diverse team that overlooks social and structural determinants of health, can delay equitable AI advancements for priority populations (12, 31, 32, 58).

### 3.3 Opportunities to strengthen health equity integration in AI use for public health

It is essential to explicitly prioritize health equity in AI technologies for public health, to adequately address issues related to social and structural determinants of health in AI (20, 21, 29, 30, 32, 34, 37, 41, 65). This includes maintaining human oversight throughout the AI lifecycle to reduce biases, system vulnerabilities, and epistemological gaps (12, 22, 34, 41, 66). Involving disproportionately impacted priority populations during the development and validation phases ensures the representation of diverse perspectives, while supporting the identification of potential biases before implementation (21, 31, 32, 35, 41, 58, 66). This approach can also provide social and economic opportunities for those affected by the “digital divide” (58).

Bias mitigation strategies include model interpretability, fairness-aware causal modeling, and incorporating social and structural determinants of health into training data when available (6, 8, 12, 20, 22, 29–32, 34, 36, 38, 41, 42, 52). Acknowledging bias when it cannot be fully mitigated ensures accountability and fosters discussion about AI’s appropriateness in various public health settings (29, 30, 42, 57).

Ethical AI governance requires standardized guidelines centered on privacy, security, and human well-being, alongside substantial investment in AI safety, research, training, and infrastructure (50). Adopting the fair, accountable, secure, transparent, educated, and relevant (FASTER) principles throughout governance structures can promote equitable AI (22, 25, 30, 42, 56, 58, 65). A centralized AI governance body, regular testing, and standardized guidelines are also critical for enhancing accountable, secure, fair, and relevant AI (38, 42, 44, 57, 58, 65, 67).

To enhance transparency, AI models should be interpretable, using techniques like decision trees and Gaussian processes to address “black-box” issues (12, 52, 58, 68). Intersectoral collaboration across sectors can promote data transparency, capacity building, strengthen peer review, and support knowledge exchange between public health professionals and AI developers (12, 30, 32, 35, 36, 38, 41, 42, 56, 58, 62, 67).

## 4 Discussion

While Canada is prioritizing AI development and implementation, it is lagging in addressing challenges related to its use in public health and across social policy more broadly (24). Biases, unrepresentative datasets, and institutional lag are barriers to integrating health equity in AI for public health. Opportunities include adopting FASTER principles to strengthen ethical, security, and privacy guidelines, enhancing AI governance, and promoting intersectoral collaboration. However, implementing these opportunities in practice remains uncertain. For example, addressing the “digital divide” in remote regions by providing AI technology and engaging with communities requires sustained technological and workforce resources, both of which are currently limited (33, 39, 45, 46). Furthermore, the environmental impact of new technologies and the difficulty of standardizing guidelines across sectors could delay progress, risking deepening inequities as AI advances.

Biases in AI are particularly concerning given that they can be introduced at any point during the AI lifecycle, influencing decisions that can exacerbate inequities (37, 41). Thomasian et al. (12) describes this as the “submerged state,” where discrimination goes unnoticed but influences key public health decisions, such as how resources are allocated, and which priority populations receive interventions. Left unaddressed, these biases could further marginalize priority populations by skewing early intervention efforts, resource distribution, and data collection. A concern is whether adopting AI may shift public health priorities, focusing more on disease control and less on addressing social and structural determinants of health and equity. Without adequate disaggregated data collection on priority populations, AI risks perpetuating existing inequities. Future research should explore how AI interacts with socio-demographic factors to ensure these variables are accurately represented in AI training datasets without compromising privacy.

The study has limitations. Not all articles were screened by two independent reviewers, introducing potential selection bias. Additionally, the academic search terms were also limited, potentially missing relevant studies. The search strategy for the evidence synthesis was restricted to studies that mentioned geographic location in Canada in the title, abstract, or keywords. This limits the generalizability of findings, as it excludes international perspectives and insights that could broaden the understanding of effective practices. Moreover, limiting this review to English-language articles may have excluded relevant French-language research, introducing potential language bias. However, some gaps were addressed by revising search terms with the help of a librarian. Furthermore, the emerging nature of the topic meant a limited number of articles met the eligibility criteria, forcing reliance on grey literature and handpicked studies, further contributing to selection bias. Despite these limitations, the study offers critical insights into the integration of health equity in AI for public health.

This review contributes to a rapidly evolving field, addressing a gap by examining health equity in AI. By adopting an intersectional lens, it offers valuable insights for public health professionals, researchers, and organizations, helping guide decision-making for developing equitable AI practices. The inclusion of both academic and grey literature enriches the analysis, capturing diverse perspectives, often absent from academic discourse. This broader approach provides a more comprehensive understanding of health equity implications in AI.

## 5 Conclusion

AI can advance public health in Canada, but health equity must be considered to avoid deepening inequities among priority populations. Key challenges include gaps in AI epistemology, biases across the AI lifecycle, the “digital divide,” unrepresentative training datasets, and ethical and privacy concerns. These issues disproportionately impact priority populations, including Indigenous Peoples, racialized communities, 2SLGBTQI+ communities, women, those living on low-incomes, youth, older adults, and those in northern, rural, and remote regions. Opportunities to strengthen equity in AI include implementing diverse frameworks, ensuring human oversight throughout the AI lifecycle, using advanced modeling techniques to mitigate biases, promoting intersectoral collaboration to develop equitable AI, and standardizing ethical and privacy guidelines to enhance AI governance. Future research should explore the feasibility of these equity-informed approaches and the impact of using disaggregated data in testing datasets to enhance AI models.
