# Supplementary material for: Integrating health equity in artificial intelligence for public health in Canada: a rapid narrative review
Source: Front Public Health. 2025 Mar 18;13:1524616. doi: 10.3389/fpubh.2025.1524616 (PMC11958991; doi:10.3389/fpubh.2025.1524616)
Supplement: Supplementary file 1 [file Data_Sheet_1.zip › Supplementary material presentation/Frontiers_Supplementary Materials_Appendix A_HE for AI in PH_14-01-2024.docx]

Supplementary Material

# Appendix A. Rapid review search strategy

The search strings were developed iteratively for Medline [Ovid] and Embase [Ovid].

## Medline [Ovid]

Ovid MEDLINE(R) ALL <1946 to June 20, 2024>

| **#** | **Searches** |
| --- | --- |
| 1 | exp Artificial Intelligence/ |
| 2 | ("artificial intelligence" or AIVI or "classification algorithm*" or "computer heuristic*" or "convolutional network*" or DALL-E or "decision support system*" or "decision tree" or DeepAI or "deep learning" or "data science" or "feature detection" or "generative pre-trained transformer" or "generative pretrained transformer" or Invideo or "language learning model*" or "large language model*" or "learning algorithm*" or "machine learning" or (Markov adj3 model*) or Midjourney or ((multifactor* or multicriteria) adj3 ("decision analysis" or "decision making")) or "natural language process*" or "nearest neighbo*" or "neural network*" or "outlier detection" or "pattern recognition" or Perplexity or "probability tree" or "random forest" or "representation learning" or Runway AI or Runway Gen-1 or "Stable Diffusion" or "support vector machine*" or "transfer learning" or "Bing chat" or ChatGPT* or "Chat GPT" or "Google* Bard" or "Google* Gemini" or "IBM Watson" or "Microsoft* Bing" or "Microsoft* Copilot" or OpenAI or "Open AI" or PathAI or "Path AI").mp. |
| 3 | ((("artificial intelligence" or AI) adj2 (chat* or generat*)) or GenAI or ((large or natural or generative or machine or deep learning) adj3 (language or text) adj3 model*) or AlexaTM or (Amazon* and Alexa) or Anthropic or Bard or Bardeen or BERT or "Bing chat" or BioGPT or BLOOM or BloombergGPT or Cerebras-GPT or ChatGPT* or "Chat GPT" or chatbot* or Chatsonic or Chinchilla or Claude or DALL-E or EinsteinGPT or Ernie or Falcon or Galactica or "Generative Fill" or "GitHub Copilot" or GLaM or "Google* Assistant" or "Google* Bard" or "Google* Gemini" or Gopher or GPT-1 or GPT-2 or GPT-3* or GPT-4* or GPTNeo or GPT-NEoX or GPT-J* or "IBM Watson" or LaMDA or LLaMA or "Megatron-Turing NLG" or "Microsoft* Bing" or "Microsoft* Copilot" or Midjourney or Minerva or NeevaAI or Nvidia or OpenAI or "Open AI" or OpenAssistant or PaLM or PanGu-E or PathAI or "Path AI" or Perplexity or "pre-trained transformer*" or "pretrained transformer*" or (Apple* and Siri) or SlackGPT or "Stable Diffusion" or StyleGAN or Synthesia or XLNet or YaLM 100B or YouChat).mp. |
| 4 | or/1-3 |
| 5 | exp Canada/ |
| 6 | (canadian* or canada* or british columbia* or alberta* or saskatchewan* or manitoba* or ontario* or quebec* or new brunswick* or prince edward island* or nova scotia* or labrador* or newfoundland* or nunavut* or northwest territor* or yukon* or toronto* or montreal* or vancouver* or ottawa* or calgary* or edmonton* or winnipeg* or first nation* or metis or inuit).tw,kf,jw,in. |
| 7 | or/5-6 |
| 8 | Residence Characteristics/ or Environment Design/ or exp Marital Status/ |
| 9 | (neighbo?rhood* or residential environment* or rural* or inner?city or housing instability or housing insecurity or housing strain or housing security or mortgage problems or foreclosure or eviction* or housing loss or home repossession* or home ownership or (repossess* adj3 hous*) or (repossess* adj3 propert*) or mortgage delinquency or mortgage arrears or mortgage debt* or overcrowding or (living adj1 (outside or inside or near* or adjacent)) or (household adj2 size) or marital status or marriage status or widow* or cohabit* or divorce* or single parent* or live* alone).mp. |
| 10 | Cultural Deprivation/ or Acculturation/ or Culture/ or Cross-Cultural Comparison/ or Cultural Characteristics/ or Cultural Diversity/ or Language/ or "Transients and Migrants"/ or exp "Emigrants and Immigrants"/ or Minority Groups/ or Minority Health/ or Prejudice/ or Racism/ or Xenophobia/ or Social Discrimination/ or exp Race Relations/ or exp Ethnic Groups/ or exp Continental Population Groups/ or Refugees/ |
| 11 | (minorit* or migration background or racial or racism or ethnology or race or ethnic* or non?English or language other than or latino* or latina* or latinx or hispanic* or whites or caucasian* or non?white or Torres Strait Islander or aboriginal or native american or inuit or eskimo or first nation* or indigenous or metis or english as a second language or foreign language).mp. |
| 12 | Occupations/ or Unemployment/ |
| 13 | (occupations or unemployment).mp. |
| 14 | exp Gender Identity/ or Women's Health/ or Sex Factors/ |
| 15 | (gender differences or sex disparit* or sex difference? or gender identity or sex role or wom#n* role? or m#n* role? or gender* role? or servicewomen).mp. |
| 16 | exp Educational Status/ or Education/ |
| 17 | (schooling or educational status or (education* adj2 level?) or ((higher or better or worse or less) adj educated) or ((higher or better or worse or less) adj level? of education)).mp. |
| 18 | Religion/ |
| 19 | religi*.mp. |
| 20 | Social Determinants of Health/ or Psychosocial Deprivation/ or Sociological Factors/ or Working Poor/ or Hierarchy, Social/ |
| 21 | (disparit* or inequalit* or inequit* or equity or deprivation or gini or concentration index).mp. |
| 22 | Socioeconomic Factors/ or Social Welfare/ or exp Social Class/ or exp Poverty/ or Income/ |
| 23 | (social class* or social determinants or social status or social position or social background or social circumstance* or socio-economic or socioeconomic or sociodemographic or socio-demographic or ses or disadvantaged or impoverished or poverty or economic level or assets index or income*).mp. |
| 24 | Social Stigma/ or Social Capital/ or Social Control, Informal/ or exp Social Support/ or exp Social Environment/ or Trust/ or Social Conditions/ or Social Isolation/ or Social Marginalization/ or Anomie/ or Social Participation/ |
| 25 | (social exclusion or (social adj (capital or cohes* or organis* or organiz*)) or (community adj3 (cohes& or participa*)) or ((neighbourhood or neighborhood) adj cohes*) or social relationships or social network* or collective efficacy or civil society or informal social control or neighbo*rhood disorder or social disorgani?ation or anomie or social support or social participation or trust or emotional support or psychosocial support or community capital or neighbo*rhood cohesion or social influence or soci*context* or soci*-context*).mp. |
| 26 | Health Status Disparities/ or Health Services Accessibility/ or Health Equity/ |
| 27 | (health*care disparit* or health care disparit* or health status disparit* or health disparit* or health inequalit* or health inequit* or medically underserved).mp. |
| 28 | or/8-27 |
| 29 | exp Public Health Practice/ or Disaster Planning/ or exp Health Education/ or exp Health Policy/ or exp Public Health/ |
| 30 | ((public health adj2 (setting? or practice? or research* or organi#ation?)) or ((communicable or infectious) adj disease control) or contact tracing or disease notification? or immuni?ation or vaccin* or mass drug administration or surveillance or ((disease? or illness* or outbreak? or injur* or death or suicide) adj3 (prevent* or predict*)) or ((disaster or emergency or pandemic or relief) adj2 (plan? or planning or preparation or prepared* or resilien* or response? or mitigation or manag* or strateg* or predict*)) or physical distancing or social distancing or quarantine? or health education or health promotion or sex education or smoking prevention or smoking cessation or weight reduction program? or healthy people program? or healthy canadian? or consumer health information or health literacy or health screening or border service?).ti,kf. |
| 31 | ((public health adj2 (setting? or practice? or research* or organi#ation?)) or ((communicable or infectious) adj disease control) or contact tracing or disease notification? or immuni?ation or vaccin* or mass drug administration or surveillance or ((disease? or illness* or outbreak? or injur* or death or suicide) adj3 (prevent* or predict*)) or ((disaster or emergency or pandemic or relief) adj2 (plan? or planning or preparation or prepared* or resilien* or response? or mitigation or manag* or strateg* or predict*)) or physical distancing or social distancing or quarantine? or health education or health promotion or sex education or smoking prevention or smoking cessation or weight reduction program? or healthy people program? or healthy canadian? or consumer health information or health literacy or health screening or border service?).ab. /freq=2 |
| 32 | (exp Policy/ or exp Government Programs/ or exp Legislation as Topic/ or exp Guidelines as Topic/ or Policy Making/) and (exp Public Health/ or exp Public Health Practice/) |
| 33 | ((policy or policies or law or laws or legislat* or position statement* or initiative* or regulat* or best practice* or (recommend* adj2 practice*) or protocol* or national or provincial or municipal or guideline* or program* or scheme* or strategic plan* or framework*) and public health).ti,kw. |
| 34 | ((policy or policies or law or laws or legislat* or position statement* or initiative* or regulat* or best practice* or (recommend* adj2 practice*) or protocol* or national or provincial or municipal or guideline* or program* or scheme* or strategic plan* or framework*) and public health).ab. /freq=2 |
| 35 | or/29-34 |
| 36 | 4 and 7 and 28 and 35 |
| 37 | (2014* or 2015* or 2016* or 2017* or 2018* or 2019* or 2020* or 2021* or 2022* or 2023* or 202401* or 202402* or 202403* or 202404* or 202405* or 2024060* or 2024061*).dt,ez,ed. |
| 38 | 36 and 37 |
| 39 | limit 38 to english |

## Embase [Ovid]

Embase <1946 to June 20, 2024>

| **#** | **Searches** |
| --- | --- |
| 1 | exp artificial intelligence/ or exp deep learning/ or exp machine learning/ |
| 2 | (AI or "artificial intelligence" or AIVI or "classification algorithm*" or "computer heuristic*" or "convolutional network*" or DALL-E or "decision support system*" or "decision tree" or DeepAI or "deep learning" or "data science" or "feature detection" or "generative pre-trained transformer" or "generative pretrained transformer" or Invideo or "language learning model*" or "large language model*" or "learning algorithm*" or "machine learning" or (Markov adj3 model*) or Midjourney or ((multifactor* or multicriteria) adj3 ("decision analysis" or "decision making")) or "natural language process*" or "nearest neighbo*" or "neural network*" or "outlier detection" or "pattern recognition" or Perplexity or "probability tree" or "random forest" or "representation learning" or Runway AI or Runway Gen-1 or "Stable Diffusion" or "support vector machine*" or "transfer learning" or "Bing chat" or ChatGPT* or "Chat GPT" or "Google* Bard" or "Google* Gemini" or "IBM Watson" or "Microsoft* Bing" or "Microsoft* Copilot" or OpenAI or "Open AI" or PathAI or "Path AI").mp. |
| 3 | ((("artificial intelligence" or AI) adj2 (chat* or generat*)) or GenAI or ((large or natural or generative or machine or deep learning) adj3 (language or text) adj3 model*) or AlexaTM or (Amazon* and Alexa) or Anthropic or Bard or Bardeen or BERT or "Bing chat" or BioGPT or BLOOM or BloombergGPT or Cerebras-GPT or ChatGPT* or "Chat GPT" or chatbot* or Chatsonic or Chinchilla or Claude or DALL-E or EinsteinGPT or Ernie or Falcon or Galactica or "Generative Fill" or "GitHub Copilot" or GLaM or "Google* Assistant" or "Google* Bard" or "Google* Gemini" or Gopher or GPT-1 or GPT-2 or GPT-3* or GPT-4* or GPTNeo or GPT-NEoX or GPT-J* or "IBM Watson" or LaMDA or LLaMA or "Megatron-Turing NLG" or "Microsoft* Bing" or "Microsoft* Copilot" or Midjourney or Minerva or NeevaAI or Nvidia or OpenAI or "Open AI" or OpenAssistant or PaLM or PanGu-E or PathAI or "Path AI" or Perplexity or "pre-trained transformer*" or "pretrained transformer*" or (Apple* and Siri) or SlackGPT or "Stable Diffusion" or StyleGAN or Synthesia or XLNet or YaLM 100B or YouChat).mp. |
| 4 | or/1-3 |
| 5 | exp Canada/ |
| 6 | (canadian* or canada* or british columbia* or alberta* or saskatchewan* or manitoba* or ontario* or quebec* or new brunswick* or prince edward island* or nova scotia* or labrador* or newfoundland* or nunavut* or northwest territor* or yukon* or toronto* or montreal* or vancouver* or ottawa* or calgary* or edmonton* or winnipeg* or first nation* or metis or inuit).tw,hw,kf. |
| 7 | (canadian* or canada* or british columbia* or alberta* or saskatchewan* or manitoba* or ontario* or quebec* or new brunswick* or prince edward island* or nova scotia* or labrador* or newfoundland* or nunavut* or northwest territor* or yukon* or toronto* or montreal* or vancouver* or ottawa* or calgary* or edmonton* or winnipeg* or first nation* or metis or inuit).jw,jx. |
| 9 | (Canada or Canadian).go,gc. and (canadian* or canada* or british columbia* or alberta* or saskatchewan* or manitoba* or ontario* or quebec* or new brunswick* or prince edward island* or nova scotia* or labrador* or newfoundland* or nunavut* or northwest territor* or yukon* or toronto* or montreal* or vancouver* or ottawa* or calgary* or edmonton* or winnipeg* or first nation* or metis or inuit).in. |
| 9 | or/5-9 |
| 10 | Demography/ or Environmental Planning/ or Marriage/ or Divorce/ or Cohabitation/ or Widow/ or exp “Single (Marital Status)”/ |
| 11 | (neighbo?rhood* or residential environment* or rural* or inner?city or housing instability or housing insecurity or housing strain or housing security or mortgage problems or foreclosure or eviction* or housing loss or home repossession* or home ownership or (repossess* adj3 hous*) or (repossess* adj3 propert*) or mortgage delinquency or mortgage arrears or mortgage debt* or overcrowding or (living adj1 (outside or inside or near* or adjacent)) or (household adj2 size) or marital status or marriage status or widow* or cohabit* or divorce* or single parent* or live* alone).mp. |
| 12 | exp Cultural Deprivation/ or Cultural Factor/ or Cultural Anthropology/ or Cultural Diversity/ or exp Migrant/ or Minority Group/ or Minority Health/ or Prejudice/ or exp Social Discrimination/ or exp Race Relation/ or exp Ethnic Group/ or exp Ancestry Group/ or exp Refugee/ |
| 13 | (minorit* or migration background or racial or racism or ethnology or race or ethnic* or non?English or language other than or latino* or latina* or latinx or hispanic* or whites or caucasian* or non?white or Torres Strait Islander or aboriginal or native american or inuit or eskimo or first nation* or indigenous or metis or english as a second language or foreign language).mp. |
| 14 | exp Employment Status/ or Job Characteristics/ |
| 15 | (occupations or unemployment).mp. |
| 16 | exp Gender Identity/ or Women's Health/ or Sex Difference/ |
| 17 | (gender differences or sex disparit* or sex difference? or gender identity or sex role or wom#n* role? or m#n* role? or gender* role? or servicewomen).mp. |
| 18 | exp Educational Status/ |
| 19 | (schooling or educational status or (education* adj2 level?) or ((higher or better or worse or less) adj educated) or ((higher or better or worse or less) adj level? of education)).mp. |
| 20 | Religion/ |
| 21 | religi*.mp. |
| 22 | “Social Determinants of Health”/ or Social Aspect/ or Working Poor/ or exp Social Hierarchy/ or Socioeconomics/ |
| 23 | (disparit* or inequalit* or inequit* or equity or deprivation or gini or concentration index).mp. |
| 24 | Social Welfare/ or Social Class/ or Poverty/ or Social Status/ or Social Background/ |
| 25 | (social class* or social determinants or social status or social position or social background or social circumstance* or socio-economic or socioeconomic or sociodemographic or socio-demographic or ses or disadvantaged or impoverished or poverty or economic level or assets index or income*).mp. |
| 26 | exp Social Isolation/ or Social Capital/ or Social Stigma/ or Social Support/ or Social Environment/ or Trust/ or exp Social Exclusion/ or Anomie/ or Social Participation/ |
| 27 | (social exclusion or (social adj (capital or cohes* or organis* or organiz*)) or (community adj3 (cohes& or participa*)) or ((neighbourhood or neighborhood) adj cohes*) or social relationships or social network* or collective efficacy or civil society or informal social control or neighbo*rhood disorder or social disorgani?ation or anomie or social support or social participation or trust or emotional support or psychosocial support or community capital or neighbo*rhood cohesion or social influence or soci*context* or soci*-context*).mp. |
| 28 | Health Disparity/ or Health Equity/ or Health Care Access/ |
| 29 | (health*care disparit* or health care disparit* or health status disparit* or health disparit* or health inequalit* or health inequit* or medically underserved).mp. |
| 30 | or/10-29 |
| 31 | Public Health/ or exp Disaster Management/ or exp Health Education/ |
| 32 | ((public health adj2 (setting? or practice? or research* or organi#ation?)) or ((communicable or infectious) adj disease control) or contact tracing or disease notification? or immuni?ation or vaccin* or mass drug administration or surveillance or ((disease? or illness* or outbreak? or injur* or death or suicide) adj3 (prevent* or predict*)) or ((disaster or emergency or pandemic or relief) adj2 (plan? or planning or preparation or prepared* or resilien* or response? or mitigation or manag* or strateg* or predict*)) or physical distancing or social distancing or quarantine? or health education or health promotion or sex education or smoking prevention or smoking cessation or weight reduction program? or healthy people program? or healthy canadian? or consumer health information or health literacy or health screening or border service?).ti,kf. |
| 33 | ((public health adj2 (setting? or practice? or research* or organi#ation?)) or ((communicable or infectious) adj disease control) or contact tracing or disease notification? or immuni?ation or vaccin* or mass drug administration or surveillance or ((disease? or illness* or outbreak? or injur* or death or suicide) adj3 (prevent* or predict*)) or ((disaster or emergency or pandemic or relief) adj2 (plan? or planning or preparation or prepared* or resilien* or response? or mitigation or manag* or strateg* or predict*)) or physical distancing or social distancing or quarantine? or health education or health promotion or sex education or smoking prevention or smoking cessation or weight reduction program? or healthy people program? or healthy canadian? or consumer health information or health literacy or health screening or border service?).ab. /freq=2 |
| 34 | (exp Policy/ or exp Health Program/ or exp Health Promotion/ or Education Program/ or Government/ or Government Regulation/) and (exp Public Health/ or exp Public Health Service/) |
| 35 | ((policy or policies or law or laws or legislat* or position statement* or initiative* or regulat* or best practice* or (recommend* adj2 practice*) or protocol* or national or provincial or municipal or guideline* or program* or scheme* or strategic plan* or framework*) and public health).ti,kw. |
| 36 | ((policy or policies or law or laws or legislat* or position statement* or initiative* or regulat* or best practice* or (recommend* adj2 practice*) or protocol* or national or provincial or municipal or guideline* or program* or scheme* or strategic plan* or framework*) and public health).ab. /freq=2 |
| 37 | or/31-36 |
| 38 | 4 and 9 and 30 and 37 |
| 39 | (2014* or 2015* or 2016* or 2017* or 2018* or 2019* or 202*).dc,dd. |
| 40 | 38 and 39 |
| 42 | limit 40 to english |

## References

Campbell SM, Kung J. Filter to Retrieve Studies Related to Artificial Intelligence from the OVID MEDLINE Database. Geoffrey & Robyn Sperber Health Sciences Library, University of Alberta. Rev March 3, 2024. <https://docs.google.com/document/d/1eWyO0jv9_6FYsxyC5LUYwFe9eH_3h83-tPNZ6wmos18/edit#heading=h.qi55eeyvgzy9>

Canada - Standard - MEDLINE, Embase, PsycInfo. In: CADTH Search Filters Database. Ottawa: CADTH; 2024: <https://searchfilters.cadth.ca/link/8>. Accessed 2024-06-03.

Development and Validation of a Search Filter to Identify Equity-Focused Studies (Stephanie L. Prady, Eleonora P. Uphoff, Madeleine Power, Su Golder)

Kung JY & Chojecki D. Filter to Retrieve Studies Related to Generative AI from the OVID MEDLINE Database. Geoffrey & Robyn Sperber Health Sciences Library, University of Alberta. Rev March 3, 2024. <https://docs.google.com/document/d/1fPiY7GmI_Z4xZQTqZ58DySUTJousLlZr1GTCP-kDOLA/edit#heading=h.qi55eeyvgzy9>
