# Supplementary material for: Integrating health equity in artificial intelligence for public health in Canada: a rapid narrative review
Source: Front Public Health. 2025 Mar 18;13:1524616. doi: 10.3389/fpubh.2025.1524616 (PMC11958991; doi:10.3389/fpubh.2025.1524616)
Supplement: Supplementary file 1 [file Data_Sheet_1.zip › Supplementary material presentation/Frontiers_Supplementary Materials_Appendix B_HE for AI in PH_23-10-2024.docx]

Supplementary Material

# Appendix B. Mapping of health equity considerations across included studies.

## *Marked as grey literature or hand-picked journal articles.

**Abbreviation: Public health (PH).

***Health equity considerations:

- Individual social identities: Examples include sex, race, language, ethnicity, income, age, (dis)ability, gender, geography, Indigenous identity, culture, religion, sexual orientation, and education.
- Group membership: Examples include family, peer groups, and social networks.
- Social context: Examples include institutions, privilege, attitudes, norms, and beliefs.
- Systems of oppression: Examples include systemic/structural inequalities, racism, sexism, ableism, ageism, classism, religious oppression, and distribution of resources and power.

| First author, publication year | Public health  Setting | Health equity considerations*** | | | |
| --- | --- | --- | --- | --- | --- |
|  |  | Individual social identities | Group membership | Social context | Systems of oppression |
| Ali Feizollah et al., 2022 | PH research |  |  | **X** |  |
| Baclic et al., 2020 | General PH |  |  | **X** | **X** |
| Bavli and Galea, 2024* | PH research; PH program |  |  | **X** | **X** |
| Berdahl et al., 2023 | General PH | **X** | **X** | **X** | **X** |
| Berridge and Grigorovich, 2022 | General PH; PH surveillance | **X** | **X** | **X** | **X** |
| Biswas, 2023* | General PH; PH Research |  |  | **X** |  |
| Borgesius, 2018* | Policy |  |  | **X** | **X** |
| Bouchouar et al., 2021 | PH research; PH surveillance |  |  | **X** |  |
| Bourgeois-Doyle, 2019* | Policy; research |  |  | **X** |  |
| Brooks, 2024* | PH research |  |  | **X** | **X** |
| Canadian Institute for Advanced Research, 2020* | Policy; research |  |  | **X** |  |
| Canadian Institute for Advanced Research, 2018* | PH research |  |  | **X** |  |
| Canadian Institutes of Health Research and Canadian Institute for Advanced Research, 2024* | General PH; PH research |  |  | **X** |  |
| Canadian Institutes of Health Research, 2019* | General PH; PH research |  | **X** | **X** | **X** |
| Chin et al., 2023 | PH research |  | **X** | **X** |  |
| Côté et al., 2024 | Research |  |  | **X** |  |
| Couture et al., 2023* | General PH |  | **X** | **X** |  |
| Dankwa-Mullan et al., 2021* | General PH |  | **X** | **X** | **X** |
| Dubay, 2024 | Research, Policy |  |  | **X** | **X** |
| Edward, 2020 | Research |  |  | **X** |  |
| Fisher and Rosella, 2022 | General PH | **X** |  | **X** | **X** |
| Fonseka et al., 2019 | PH research, PH program |  |  | **X** | **X** |
| Gilbert et al., 2020* | General PH; PH surveillance, PH research |  |  | **X** |  |
| Gómez-Ramírez et al., 2021 | General PH |  | **X** | **X** |  |
| Government of Canada, 2024* | Policy |  |  | **X** | **X** |
| Gurevich et al., 2022 | General PH |  |  | **X** | **X** |
| Hernandez, 2024* | PH research |  |  | X | X |
| Jang et al., 2021 | PH research |  |  | X |  |
| Kamyabi et al., 2024 | General PH |  | X | X | X |
| Kaushal et al., 2023 | PH research |  |  | X |  |
| Kharrat et al., 2024 | PH program; PH research |  |  | X |  |
| Kleiner, 2024* | Research |  |  | X |  |
| Koohsari et al., 2022 | General PH | X |  | X |  |
| Kung, 2019* | Research |  | X | X |  |
| Kung, 2021* | Research |  | X | X | X |
| L’Allié and Tran, 2023* | Policy |  |  | X |  |
| Luccioni and Bengio, 2020* | Policy; research |  | X | X | X |
| Mhasawade et al., 2021 | General PH; PH research | X | X | X | X |
| Mila, 2024* | Policy; research |  |  | X |  |
| Millar et al., 2019* | Policy; research |  |  | X |  |
| Morgenstern et al., 2021* | General PH |  |  | X | X |
| New Frontiers in Research Fund, 2023 | Research |  |  | X | X |
| Parson et al., 2019* | Policy; research |  |  | X |  |
| Rilkoff et al., 2024 | PH surveillance |  |  | X | X |
| Rolnick, 2024* | Research |  | X | X |  |
| Smith et al., 2020 | General PH |  |  | X | X |
| Statistics Canada, 2023* | Policy |  |  | X |  |
| Stobbe, 2023* | Policy |  |  | X |  |
| Thomasian et al., 2021 | General PH, PH policy |  | X | X | X |
| Vishwanatha et al., 2023* | PH research |  | X | X | X |
| Warin, 2021 | PH research, PH policy |  |  | X |  |
| World Health Organization, 2021* | General PH; PH surveillance |  |  | X | X |
| Yip et al., 2024* | General PH; PH research |  |  | X | X |
| Zhou et al., 2023 | PH research, PH surveillance |  |  | X |  |
